# Congenital *Plasmodium vivax *malaria mimicking neonatal sepsis: a case report

**DOI:** 10.1186/1475-2875-9-63

**Published:** 2010-03-01

**Authors:** Veronica Del Punta, Maurizio Gulletta, Alberto Matteelli, Vania Spinoni, Antonio Regazzoli, Francesco Castelli

**Affiliations:** 1 Institute of Infectious and Tropical Diseases, University of Brescia, Piazza Spedali Civili, 1 25125 Brescia, Italy; 2 Department of Neonatology and Neonatal Intensive Care, Spedali Clivili, Brescia, Italy; 3 Laboratory of Chemical and Clinical Analysis, Spedali Civili, Brescia, Italy

## Abstract

Although malaria in pregnancy can cause very significant neonatal morbidity, congenital malaria is a very rare condition in both endemic and non-endemic areas. A case of congenital malaria by *Plasmodium vivax*, initially mistaken for neonatal sepsis, is described. The correct diagnosis was accidentally done, as congenital malaria had been missed in the initial differential diagnosis.

Vivax malaria is the leading species in congenital infections in Europe. This condition should be included in the differential diagnosis of neonatal sepsis even if the mother has no proven malarial episodes during the gestational period.

## Background

Both *Plasmodium falciparum *and *Plasmodium vivax *infections can cause adverse pregnancy outcomes, including maternal anaemia, low birth-weight due to preterm delivery and foetal growth restriction [1]. Pregnant women are more susceptible than non-pregnant women to malaria, especially in first and second pregnancy [2]. On the contrary, congenital malaria remains extremely rare both in endemic and non-endemic areas [3]. In endemic countries congenital malaria is mainly caused by *P. falciparum *[4]. In European countries most cases are due to *Plasmodium malariae *and *P. vivax *[5], associated with the decrease over time of malaria immunity, and the immunosuppression observed at the end of pregnancy [5].

A case of congenital malaria by *P. vivax *is presented and its differential diagnosis with other forms of neonatal sepsis are discussed.

## Case presentation

A 22-day-old female infant delivered from a Pakistani mother was referred to the Department of Neonatology and Neonatal Intensive Care, Spedali Civili, Brescia, for intermittent fever of one week duration with picks above 39 C°. The mother had a spontaneous, eutocic delivery at 40 week gestation with no abnormal events during the pregnancy period. The newborn birth weight was 2750 gr. She had mixte feeding and, up to three days before admission, she had been in good health.

On admission, the infant was in bad general conditions. Physical examination revealed paleness, whining cry, liver and spleen enlargement. Breathing and cardiac functions were normal. Laboratory evaluation showed mild anaemia (haemoglobin Hb: 12.3 g/dl), normal red and white blood cell counts (RBC 3.620.000/mmc, WBC 4.960/mmc) and severe thrombocytopaenia with platelets (PLT) 14.000/cmm. Other relevant results included mild hyper-bilirubinaemia: 4.14 mg/dl, hyper-transaminasaemia: aspartate aminotransferase 494 U/l and elevated C-reactive protein (CRP) at 73.2 mg/dl (n.v. <5 mg/dl). The remaining biochemistry parameters and metabolic values were within normal limits. Blood and urine cultures were negative. A clinical diagnosis of neonatal sepsis was done and antibiotic treatment consisting of intravenous ampicillin and gentamicin was started.

Twenty-four hour after admission, by accidental examination of the peripheral blood to determine the differential white blood cell count, haemoparasites were noted within red blood cells. A thick and thin blood film revealed *P. vivax *trophozoites and few schizonts, with a parasitaemia of 2%. The diagnosis of vivax malaria was eventually confirmed by polymerase chain reaction (PCR).

The infant was administered oral chloroquine 10 mg/kg the first and the second day followed by 5 mg/kg the third day. Four days after treatment peripheral parasitaemia was completely cleared, the white blood cell count increased up to 13.180/mmc, and the CRP fell down to 10.5 mg/dl.

The infant was discharged on day five with normal red and white blood cell counts, haemoglobin value, platelets count, biochemical profile, and normal inflammatory index (CRP < 5 mg/dl). No recrudescence was seen after six months of follow up.

The history of the mother was re-evaluated. She was a primigravida. She reported one clinical malaria attack in August 2008 (before becoming pregnant) during a short stay in Pakistan to visit relatives and friends. She reportedly received treatment with sulphadoxine-pyrimethamine. In May 2009, when she was at 32 week gestation she had been admitted to hospital in Italy for fever, anaemia (Hb 11.3 mg/dl,) and thrombocytopaenia (PLT 97.000/mmc). She received a diagnosis of urinary tract infection, was treated with amoxicillin/clavulanic acid, and she was discharged after four days of hospital stay.

The mother was re-evaluated at the light of the newborn diagnosis. She had no clinical symptoms of malaria and her blood smear thick film was negative for malaria parasites. No antimalarial drugs for either the haematic or liver stages were administered.

## Discussion

This case shows that the diagnosis of congenital malaria should be considered as an important differential diagnosis of neonatal sepsis in infants who are born from mothers coming from malaria endemic countries with or without a history of malarial disease during pregnancy. The epidemiological concept of malaria exposure is of obvious paramount importance in this context. However, a proven history of malaria episodes of the mother during the gestational period is not essential: the episode may be mild enough not to require medical attention, or, as it most likely occurred in the case described, may go undiagnosed during a careless diagnostic procedure.

Postulated mechanisms for congenital transmission of malaria parasites include maternal transfusion into the foetal circulation either at the time of delivery or during pregnancy, direct penetration through the chorionic villi, or penetration through premature separation of placenta [6]. The remarkable capacity of the foetus to resist infection has been demonstrated [7]. This resistance can reflect the physical barrier of the placenta to infected red cells, the passive transfer of maternal antibodies, and the unfavourable environment offered by foetal erythrocytes for plasmodial replication due to their foetal haemoglobin composition and low free-oxygen tension [6,8].

Congenital malaria can occur despite the absence of any evidence of active malaria infection of the mother during pregnancy. It can be speculated that the mother had a recrudescence episode of vivax malaria during the third trimester, which was mild, resolved spontaneously, and remained undiagnosed. Consistently with the case reported here, *P. vivax *malaria is reported to be more common in primigravidae than in multigravidae and is usually associated with mild maternal anaemia and increased risk of low birthweight [1]. Moreover, vivax malaria is not associated with miscarriage, stillbirth or with a shorten duration of pregnancy [1]. In this case, the mother presented mild anaemia (Hb 11.3 mg/dl) and the infant was delivered spontaneously at term with a normal birth weight. The time of onset of clinical symptoms in congenital malaria can vary from immediately after birth to ten weeks, but several studies underline that the median age of manifestation is 21 days [3,9].

Diagnosis is simple with microscopic examination of blood films, though a clinical suspicion of the disease is required to start an appropriate diagnostic procedure. This report underlines that diagnosis can be achieved by accidental observation of parasites in a blood film, even when the diagnosis had not been previously suspected.

The clinical features of neonatal malaria include anaemia (77%), fever (74%), liver and spleen enlargement (68%), poor feeding/lethargy/irritability and jaundice [10-12]. Severe thrombocytopaenia without bleeding, is also a frequently reported feature of congenital malaria [5,9,13].

The treatment of congenital *vivax *malaria requires a blood schizonticide, like chloroquine, whereas primaquine is unnecessary as in congenital malaria there is no hepatic stage of the parasite.

The higher prevalence of congenital malaria due to *P. vivax *than to *P. falciparum *in non-endemic countries is well established [1,5]. The most likely explanation is represented by the longer incubation time and the relatively milder clinical presentation in *P. vivax *malaria, which allows for more maternal episodes to go undiagnosed and untreated. A potential additional determinant is represented by the contraindication during pregnancy of drugs that can eradicate the liver stage of the parasite, thus increasing the likelihood of late relapses.

In conclusions, *P. vivax *congenital malaria is a rare condition that should be included in the differential diagnosis of neonatal sepsis in newborns at epidemiological risk. More studies are needed to assess the mechanism of maternal transmission of *P. vivax *malaria, and its incidence in both endemic and non-endemic countries.

## Competing interests

The authors declare that they have no competing interests.

## Authors' contributions

DPV provided clinical care for the case and wrote the manuscript

GM, MA, and CF provided clinical parasitological consultation and revised the manuscript

SV provided neonatological consultation and revised the manuscript

RA was responsible for diagnostic investigation and revised the manuscript

All authors read and approved the final manuscript.
